# Bacterial RNA chaperones and chaperone-like riboregulators: behind the scenes of RNA-mediated regulation of cellular metabolism

**DOI:** 10.1080/15476286.2022.2048565

**Published:** 2022-04-19

**Authors:** Kai Katsuya-Gaviria, Giulia Paris, Tom Dendooven, Katarzyna J. Bandyra

**Affiliations:** a Department of Biochemistry, University of Cambridge, Tennis Court Road, Cambridge CB2 1GA, UK; b Department of Structural Studies, MRC Laboratory of Molecular Biology, Francis Crick Avenue, Cambridge, CB2 0QH, UK; c Biological and Chemical Research Centre, Department of Chemistry, University of Warsaw, 02-089 Warsaw, Poland

**Keywords:** RNA chaperone, ProQ, CsrA, RNA metabolism, C-terminal domain, Hfq, Riboregulation

## Abstract

In all domains of life, RNA chaperones safeguard and guide the fate of the cellular RNA pool. RNA chaperones comprise structurally diverse proteins that ensure proper folding, stability, and ribonuclease resistance of RNA, and they support regulatory activities mediated by RNA. RNA chaperones constitute a topologically diverse group of proteins that often present an unstructured region and bind RNA with limited nucleotide sequence preferences. In bacteria, three main proteins – Hfq, ProQ, and CsrA – have been shown to regulate numerous complex processes, including bacterial growth, stress response and virulence. Hfq and ProQ have well-studied activities as global chaperones with pleiotropic impact, while CsrA has a chaperone-like role with more defined riboregulatory function. Here, we describe relevant novel insights into their common features, including RNA binding properties, unstructured domains, and interplay with other proteins important to RNA metabolism.

## Introduction

The world of cellular RNAs is surprisingly rich in complexity and regulatory potential, with RNA molecules serving indispensable roles in sophisticated mechanisms controlling cellular homeostasis and adaptive responses. Starting with stable RNAs that support translation, like rRNAs and tRNAs, through mRNAs carrying information about proteins sequence, RNA species can guide or catalyse chemical reactions, and many encompass regulatory functions in controlling the expression of genetic information. Different RNAs have explicitly defined functions in the cell, with precisely specified fold, targets, and half-life. Although the pool is immensely diverse, the individual RNA species share in common protein companions that stand by and ensure their stability and facilitate their functions.

**Figure 1.**
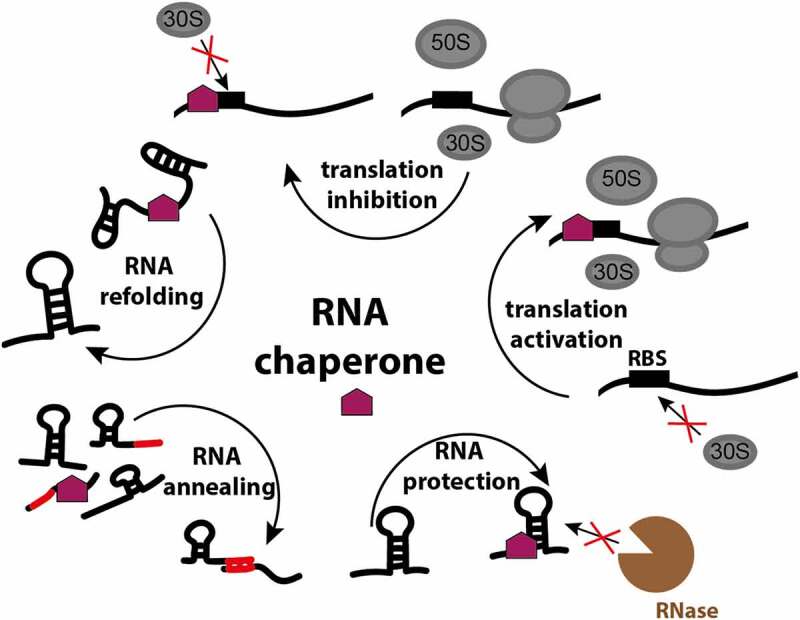
**RNA chaperone functions in the bacterial cell**. RNA chaperones facilitate proper RNA folding, remodel existing states, assist in matchmaking between regulatory RNAs and their targets, protect bound RNA from ribonucleases, and by the interaction with the ribosome-binding site (RBS) activate or inactivate translation.

RNA chaperones, the proteins that ensure RNA integrity, structure and function, are less diverse than the RNA molecules themselves, and a single chaperone is often capable of interacting with many different RNAs. In bacteria, RNA chaperones facilitate proper RNA folding, remodel existing states and expose crucial regulatory elements to assist in matchmaking between regulatory RNAs and their targets. Chaperones also protect bound RNA from ribonucleases and effectively steer RNA molecules to their destination (Fig. 1). As such, chaperones participate in many RNA-mediated processes, and constitute an integral part of many pathways involving RNA-mediated regulation. To support such a broad interactome, chaperones must possess fairly promiscuous traits. For example, many chaperones present unstructured regions that allow for comparatively faster accommodation of diverse substrates and for binding events where specificity is effectively uncoupled with interaction strength [1,2].

The biological necessity of RNA chaperones might be apparent when considering that RNAs are highly labile molecules and do not exist naked in the cell due to the high probability of rapid degradation by ribonucleases. In addition, RNAs are postulated to be prone to misfolding, and incorrect structures of RNA molecules can lead to significant inefficiency of RNA-dependent processes or aggregation [3]. Various RNA-binding proteins like helicases and adaptor proteins can have chaperoning activity, resolving trapped folding intermediates simply via interaction with their specific partner RNA molecules. Ribosomal proteins S1 and S12 have also been described as chaperones, having a mixture of properties of specific (ribosome formation) and non-specific (interaction with non-rRNA) chaperones [4,5]. However, bacteria possess an additional class of specialised chaperone or chaperone-like proteins, including Hfq, ProQ, CsrA and cold shock proteins (CSPs), that interact with a multitude of RNA types and help to confer integrity of the total cellular RNA pool [6]. Here, we summarise the characteristics of the best studied bacterial RNA chaperones Hfq and ProQ, and the chaperone-like CsrA and describe relevant recent findings regarding their mechanism of action and role in RNA metabolism.

## Bacterial RNA chaperones: who, how and where*Hfq, the most recognised RNA chaperone*

One of the most conserved and pleiotropic RNA chaperones in bacteria is Hfq. First described as an essential host factor of the RNA bacteriophage Qβ, Hfq was quickly recognised as an indispensable element of bacterial RNA metabolism. Hfq belongs to the widely occurring family of Sm and Sm-like (LSm) proteins, which places its evolutionary origins as far back as the common ancestor of bacterial, eukaryotic and archaeal lineages [7]. Hfq has been shown to influence numerous complex bacterial phenotypes, such as growth rates and yields, cell size, osmosensitivity, oxidation of carbon sources and sensitivity to ultraviolet light [8], stress response and virulence [9]. At present, Hfq is perceived as one of the main players in post-transcriptional regulation of gene expression in Enterobacteriaceae, where it aids regulation of transcripts through direct interaction, as well as by stabilising small, non-coding RNAs (sRNAs) and matching them with their RNA targets [7,10].

Hundreds of sRNAs have been reported to interact with Hfq which assists sRNA-mediated regulation through several mechanisms [7]. Upon binding to the RNA chaperone, sRNAs gain protection from ribonuclease cleavage. Then, Hfq-bound sRNAs can pair with their target mRNA and suppress its translation when the binding renders the 5’ region of the mRNA inaccessible for the protein synthesis machinery. Conversely, Hfq-sRNA complexes can promote translation when they disrupt elements of secondary structure in mRNAs that would otherwise block the access of ribosomes. Additionally, sRNA-mRNA duplex formation may induce mRNA cleavage by ribonucleases, whereas the sRNA can follow the same fate or be recycled for multiple rounds of gene expression reprogramming [11]. Finally, Hfq may bind to an mRNA and induce its polyadenylation by poly(A) polymerase triggering 3’ to 5’ degradation of the RNA molecule. Besides its implications in post-transcriptional regulation, recent reports have described a role for sRNA-mediated regulation at a co-transcriptional level [12–14]. Hfq-sRNA complexes can interact with nascent transcripts to yield their 5’ UTRs inaccessible to Rho termination factor, thus preventing premature termination and activating the expression of certain genes [12]. Conversely, Hfq-mediated sRNA binding to a target mRNA can also promote Rho-dependent termination as shown for the ChiX sRNA regulation of the *chiPQ* operon in *Salmonella* [14].

### Phylogenetic distribution

About half of bacterial genomes encode Hfq or its close homologues [15], and functionally equivalent proteins with weak homology to Hfq have also been described in some bacterial species including Cyanobacteria [16]. Hfq has been most extensively studied in gram-negative bacteria, like *Escherichia coli* and *Salmonella enterica*, but is also present in some gram-positive bacteria, where it participates in stress response and virulence [17,18]. Hfq is found in α, β, γ and proteobacteria classes with a few exceptions of species with reduced genome size associated with parasitic lifestyle [14] (Table 1). It was suggested that the presence of Hfq correlates with the GC content of the bacterial genome, as GC enriched transcripts would produce more stably interacting RNAs that require a chaperone to form productive complexes and circumvent trapping by random pairing. This correlation would explain the necessity of Hfq for sRNA action in the majority of γ-proteobacteria, in which the overall genome GC content is 50–67%. However, some bacteria possessing low GC content genomes still encode this protein [19]. Among the gram-positive bacteria with low GC content genomes that still express Hfq are *Staphylococcus aureus, Neisseria monocytogenes*, and some clades among *Bacillus* and *Clostridium* genera [14]. The role of Hfq in *Staphylococcus aureus* is not clear as the deletion of the chaperone does not impair sRNA mediated regulation in this bacterium [19–21]; however, its function could be redundant or different than in *E. coli*.

**Table 1.** **Distribution of RNA chaperones and riboregulators in Bacteria, and representative species for the groups**. Hfq, ProQ and CsrA have not been described in all species within these groups

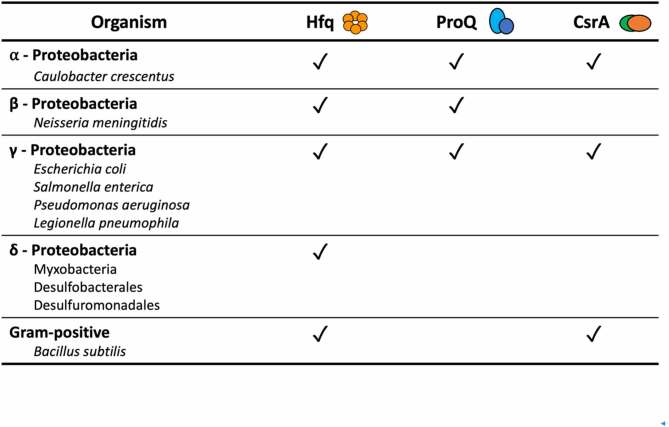

### Structure and interaction with RNA

Sm-like proteins, such as Hfq, are characterised by a ring-like, multimeric quaternary architecture. The homohexameric ring-shaped Hfq core is formed by two Sm folds conserved among Hfq homologues: Sm1, an α-helix followed by three β-strands, and the subsequent Sm2, two β-strands forming antiparallel β-sheets with Sm1. The unstructured C-terminal tail of the protein that protrudes from each protomer is poorly conserved or even absent in some bacterial species [14,22].

**Figure 2.**
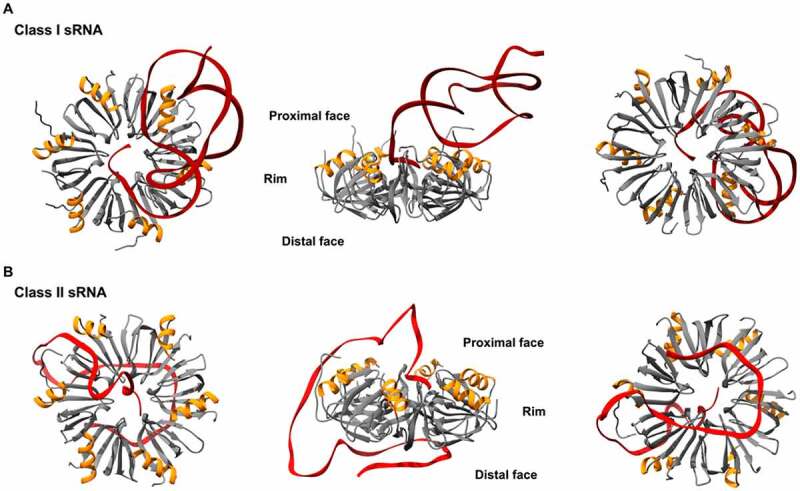
**Structures of *E. coli* Hfq-RNA complexes**. The hexameric architecture of Hfq (grey) exposes three RNA binding surfaces: proximal face (characterised by the N-terminal α-helix, shown in orange), rim and distal face. Three views of the Hfq-RNA complexes are shown, with the proximal face (left), the rim (centre), and the distal face (right) of Hfq in the foreground. (A) class I sRNAs interact with Hfq through the proximal face and the rim. Here, the *E. coli* Hfq-RydC complex is used as a model of this mode of binding (PDB: 4V2S). (B) Class II sRNAs bind to the proximal and distal faces of Hfq. *E. coli* Hfq-3’ETS^leuZ^ have been isolated from a ternary complex with PNPase to depict this mode of binding (PDB: 7OGM).

The core of Hfq forms a platform for RNA interaction with three RNA binding surfaces available, each with different affinities for specific RNA sequences (Fig. 2). The proximal face, where the amino-terminal alpha-helix is exposed, shows preference for poly uridine sequences, which are enriched at the sRNAs 3’ ends. The distal face, which is opposite to the proximal face, binds sequences with ARN-repeat motifs (where A stands for adenine, R for purine and N for any base). The rim shows preference for A/U rich sequences [23–25]. Available structures of Hfq in complex with RNA explain how the chaperone architecture influences the RNA sequence preferences: from the uridine binding pockets on the proximal face [23,26], through the central cavity on the distal site that can accommodate AR bases from the ARN motif [25,27] and, transiently, the N base in a putative pocket [28]; to the conserved arginine residues at the rim of the chaperone that form electropositive patches attracting A/U rich stretches in RNAs [24,26]. The rate of Hfq-mediated RNA annealing appears to be determined by the size of these arginine patches [29]. Whereas *E. coli* Hfq and *Pseudomonas aeruginosa* Hfq present arginine patches with three (-RRER-) and two (-RKER-) arginine residues, respectively, Hfq variants in some gram-positive bacteria with diminished RNA annealing activity, like *Bacillus subtilis* Hfq (-RKEN-) or *S. aureus* Hfq (-KANQ-), have lower arginine content.

sRNAs have different modes of binding to Hfq and are generally classified into two groups – Class I or Class II – based on their proposed modes of interaction [10,30]. Class I sRNAs interact with the proximal face of Hfq, through U-rich stretches at the 3’ end, and with the rim of the chaperone, via U/A-rich regions [23,24]. Their targets (named accordingly Class I mRNAs) have (ARN)n motifs through which they can bind to the distal face of Hfq [31]. In contrast, Class II sRNAs bind to the proximal and distal faces of Hfq through U-rich and (ARN)n motifs, respectively, and their targets Class II mRNAs interact with the rim through U/A rich motifs [10,32].

### Cellular localisation

In exponentially growing *E. coli* cells, Hfq localises predominantly in the cytoplasm, while 10–20% of the molecules can be found in the nucleoid region [33,34]. Hfq is also localised in the vicinity of the inner membrane, where it can cooperate with the main bacterial ribonuclease RNase E and other components of the RNA degradosome, a multi-protein complex responsible for RNA metabolism, to determine the post-transcriptional fate of certain RNA molecules [35–37]. Single-particle tracking studies have shown that Hfq diffuses freely in the absence of stress and that a majority of the Hfq proteins are mRNA-bound during exponential growth [34].

However, when *E. coli* cells are subjected to certain stresses, which often provoke an sRNA-mediated response, the diffusivity and cellular distribution of Hfq changes. Recent studies have shown that Hfq and sRNAs accumulate at the poles of *E. coli* cells when these are subjected to hyperosmotic stress [38]. Levels of mRNAs after a hyperosmotic shock increase or decrease depending on whether they are positively or negatively regulated, respectively, by their cognate sRNAs, and the formation of the Hfq foci might facilitate this globally acting emergency stress response.

Another strong trigger of stress response is loss of nitrogen nutrient availability. Under nitrogen starvation, *E. coli* cells experience growth attenuation and activate the nitrogen regulation stress response [39]. While cells can survive prolonged periods of nitrogen starvation and resume growth when nitrogen becomes available, the ability to adapt to this stress is highly compromised in the absence of Hfq [40]. Under conditions of nitrogen depletion, Hfq molecules gradually cluster into single large (~0.3 μm) foci which are also predominantly, but not exclusively, located at the cell poles [40]. These are reversible and dissipate upon replenishment of nitrogen. While the behaviour of Hfq molecules under nitrogen starvation is comparable to what can be observed in cells subjected to hyperosmotic stress, Hfq foci do not form under other stress conditions, such as prolonged carbon starvation or in cells in late stationary phase [40]. Thus, the accumulation patterns of Hfq might have a specific role in the adaptation to certain kinds of stress. Moreover, in nitrogen starved bacteria, RNase E forms a single and large focus, that colocalises with that of Hfq, forming ‘H-bodies’, which resemble subcellular assemblies that form by liquid-liquid phase separation [38,41]. Hfq foci formation appears to be independent of the interaction of the chaperone with RNase E, but RNase E foci formation is abolished in Δ*hfq* bacteria. Overall, these findings suggest a compartmentalisation of ribonucleoprotein complexes, in which Hfq plays a central role, involved in the adaptative response to certain stresses through post-transcriptional regulation of gene expression.

### Competition and cooperativity in Hfq–RNA interaction

Hfq is an abundantly expressed protein, with levels ranging 3,000–10,000 Hfq hexamers in *E. coli* cells depending on the growth conditions [33,42]. However, binding-competent RNAs are in molar excess, so that a majority of the chaperone molecules are predicted to be RNA-bound during exponential growth [34,43–47]. Different studies have focused on the competition between RNA molecules for the binding to Hfq showing that certain RNAs can outcompete others [32,45,48]. For instance, it is generally accepted that Class II sRNAs have stronger interactions with Hfq and can outcompete Class I sRNAs [10,32]. Through their double interaction with the proximal and distal faces of Hfq, Class II sRNAs can also displace Class I mRNAs, predicted to interact with the distal face of Hfq [34]. Conversely, Class I sRNAs can co-occupy Hfq molecules bound to Class I mRNAs [34].

Hfq is known to mediate the imperfect base-pairing between sRNAs and their targets. In this regard, the chaperone could be aiding to surpass a kinetic barrier that otherwise prevents the duplex formation. Such kinetic barrier can be imposed by elements of secondary structure, like stem loops, present close to the sRNA binding site in the target mRNA. These can affect the speed of target recognition by the sRNA as well as the rate of successful RNA duplex formation [32]. Recent experiments using single-molecule Förster resonance energy transfer (smFRET) to study the kinetics of the sRNA-mRNA annealing have shown that when a sRNA base pairs to a structured mRNA, intermediate complexes form which can either be short-lived and lead to the dissociation of cognate RNAs, or induce a conformational change in the mRNA that enables the formation of a stable duplex [49]. Here, an mRNA was immobilised on a surface by fusion with a DNA moiety carrying Cy5 fluorophore. Upon addition of Hfq and Cy3-labelled sRNA, annealing was measured by the energy transfer between the donor Cy3 and the acceptor Cy5 fluorophores, which depends on the distance between them. Stronger Hfq–sRNA interactions, like those achieved by Class II sRNAs, can reduce the rate of abortive annealing [49]. Moreover, for some transcripts, Hfq–mRNA interactions seem to be insufficient and the required restructuring of stem loops present in the mRNA only appears to occur upon sRNA binding [32]. Conversely, the sole action of Hfq has been shown to be sufficient to unfold secondary structures present in certain mRNAs such as *rpoS* and *dgcM* [50,51].

Changes in the levels of sRNAs do not affect all their mRNA targets in the same way. In a recent study, Faigenbaum-Romm *et al*. evaluated the impact of overexpression of well characterised sRNAs on their respective target mRNAs, through transcriptomic analysis combined with the application of RIL-Seq (RNA interaction by ligation and sequencing) to determine the sRNA-mRNA pairs bound to Hfq under those experimental conditions [52]. The RIL-Seq strategy takes advantage of the proximity of the interacting RNAs in the ternary complex with Hfq to ligate the ends of sRNAs and their mRNA targets, and then includes high-throughput sequencing of the chimeric RNAs [46]. Their results revealed that mRNA regulation by a sRNA does not rely solely on their relative expression levels but may depend highly on the ability of the target to occupy Hfq. Some mRNA targets, however, show high Hfq occupancies and yet no significant changes in their abundance after the overexpression of their cognate sRNAs. sRNA-mediated regulation for some of these mRNA targets is presumed to act at a translation level as shown by ribosome profiling studies [53]. Nevertheless, a further set of target mRNAs that highly occupy Hfq, seems to remain unaffected at both the RNA stability and the translation levels, upon overexpression of their cognate sRNAs, and could have roles yet to be described. These observations depict a highly complex level of interplay between sRNAs and their target RNAs, and studies like the ones described above will further illuminate the control of these regulons.

## ProQ, a more recently ascertained RNA interactor

ProQ is a FinO-like protein that was initially identified as a factor affecting post-translational activation of the osmoregulatory transporter ProP [54,55]. FinO protein, a ProQ paralogue encoded in IncF plasmids, is a chaperone itself, participating in antisense RNA FinP-mediated regulation of the mRNA *traJ* [56]. Another member of the FinO domain-containing protein family, the chromosome encoded RocC (repressor of competence chaperone), has been found to assist trans-acting sRNAs involved in the regulation of bacterial competence in the human pathogen *Legionella pneumophila* [57]. ProQ was appreciated as a more general RNA chaperone after Smirnov *et al*. discovered its homologues are present in many α, β and γ proteobacteria that do not encode ProP [58] (Table 1). ProQ deletion affects expression levels of many transcripts, involved in different physiological processes, and the chaperone itself is expressed constitutively at levels comparable to Hfq and other bacterial proteins with more general functions, like ribosomal protein S1, suggesting a more global role [58]. Interestingly, currently known bacteria species lacking Hfq homologues do not encode FinO-like proteins either, suggesting a complementary role for these chaperones [59].

### Structure

**Figure 3.**
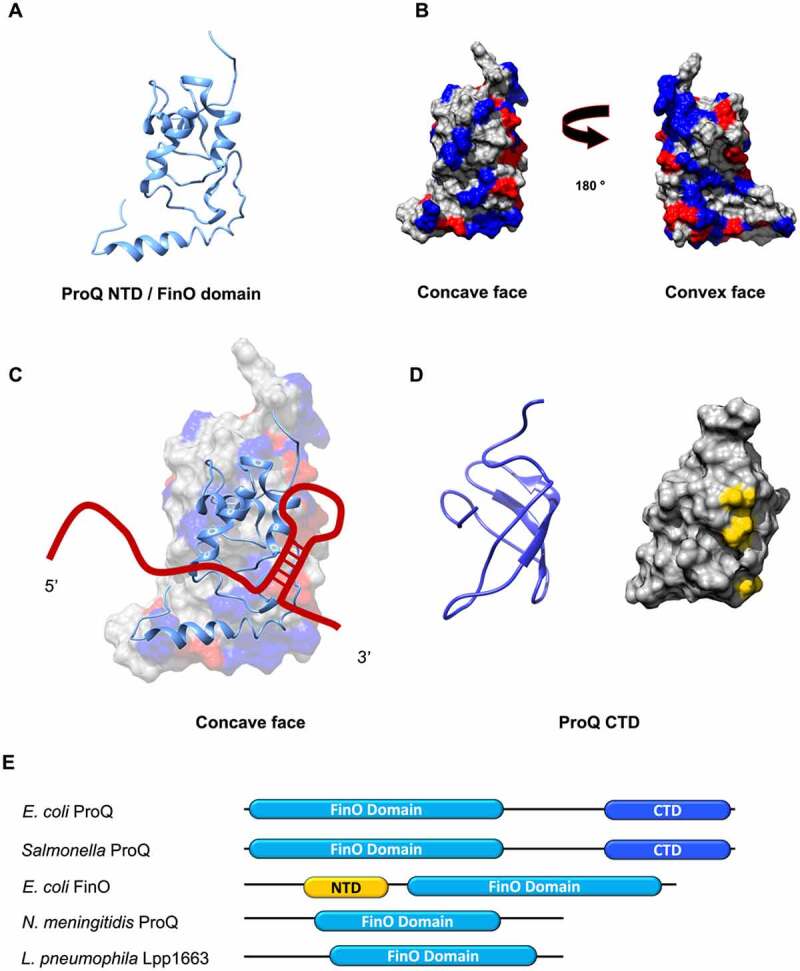
**Structure and model for RNA binding of the N-terminal domain of *E. coli* ProQ**. (A) N-terminal domain (NTD) of *E. coli* ProQ, which has the fold of the FinO domain (PDB: 5NB9). (B) ProQ NTD can be divided into two faces: a concave face, and a convex face. The concave face is RNA binding, as it presents patches of positively (in blue) and negatively (in red) charged residues. Positively charged patches on the concave face are conserved in other FinO proteins. The convex face also presents smaller areas of both negatively and positively charged residues. (C) Model of RNA binding to the NTD of ProQ. Due to the larger patches of positive residues, the concave face of the finO-domain of ProQ is expected to be mainly involved in the binding to the Rho-independent terminator hairpin at the 3’. The convex face could interact with single stranded regions nearby the double stranded hairpin [61]. (D) Structure of the CTD of *E. coli* ProQ (PDB: 5NBB). The C-terminal domain has a Tudor-domain like structure. On the right panel, the three residues G189, T204 and G220 (in *E. coli*) that have been found as important for ProQ function in gene expression regulation are highlighted in yellow. These residues are exposed on the surface of the domain and are conserved in homologous proteins [62]. (E) Distribution of the CTD of ProQ. The CTD is less conserved compared to the FinO-like NTD, as it is found only in γ-proteobacterial ProQ. In *E. coli* and *Salmonella* ProQ, the CTD is connected to the N-terminal FinO-like domain via a flexible 50 amino acids linker. The CTD is not found in the FinO protein, and it is expected to expand the ProQ interactome, by conferring it with ability to bind different RNA substrates [66].

ProQ and its homologues were identified in proteobacterial genomes and their mobile elements, like plasmids and phages. ProQ is a monomer, and it is composed of an N-terminal domain (NTD) and a C-terminal domain (CTD), connected by a flexible linker of 50 amino acids which might contribute to RNA binding [60]. The NTD of ProQ has the fold of the well characterised FinO domain (Fig. 3A), which is highly conserved and forms two faces: a concave face presenting patches of positively charged residues surrounded by negatively charged ones, and a convex face, which in the *E. coli* protein is dotted with positive and negative patches (Fig. 3B). The presence of positively charged patches on the concave face is conserved in other FinO proteins. The N-terminal domain is involved in the binding of RNA and has been proposed to recognise RNA structural elements and accommodate an RNA duplex [58–61].

The C-terminal domain of *E. coli* ProQ is mainly composed by β -strands that form a barrel-like structure, and the surface is overall electrostatically neutral [59,62] (Fig. 3D). The NMR structure of the CTD revealed a Tudor-domain fold, which is typically found in Eukarya: this Tudor-like domain could have been acquired by horizontal gene transfer, or it might have arisen by gene duplication and subsequent independent domain evolution [60]. The function of the C-terminal domain of ProQ, both in the context of RNA binding and the role of ProQ in gene regulation, is poorly understood. Moreover, it is not as conserved as the FinO-like N-terminal domain and has only been found in ProQ proteins of γ-proteobacteria (Fig. 3E).

### Interaction with RNA

Insights into ProQ-RNA interactions came from UV crosslinking and immunoprecipitation experiments followed by RNA-seq (CLIP-seq), which mapped for the first time the *in vivo* transcriptome-wide interaction of ProQ in *Salmonella enterica* and *E. coli* [63]. ProQ recognises structural features of mRNA 3’UTRs and 3’ ends of sRNAs through its N-terminal FinO domain [63,64]. The concave face of the ProQ FinO domain is the primary recognition site for RNA targets *in vivo*, with the NTD and the first 12 amino acids of the linker being sufficient for the binding [61]. The CTD does not appear to participate in RNA-binding. The concave face could be responsible for binding to RNA double stranded structures, and the convex face has been proposed to interact with single stranded regions in proximity of the RNA duplex region [61] (Fig. 3C). Mutations on the NTD RNA-binding surface cause lower intracellular levels of ProQ due to a quality control pathway that prevents cellular accumulation of defective ProQ: such molecules, as well as defective Hfq, are eliminated by the protease Lon [62,65].

One of the ProQ-binding anchors is the Rho-independent transcription terminator, with two elements required for the interaction: an oligo-U sequence at the 3’ of the hairpin, with a minimum of four uridine residues, and the double-stranded region preceding this U-stretch, since either the disruption of the base-pairing or the shortening of the oligoU stretch decrease or completely abolish ProQ binding [64]. Recent chemical shift perturbation experiments confirmed the ProQ binding preference for U-rich regions in the vicinity of a stem loop structure [66]. ProQ binding to hairpin terminator structures closely resembles the binding of Hfq to 3’ hairpins followed by polyU tails. However, ProQ and Hfq bind mainly to separate RNA pools. These RNA chaperones seem to have different preferences regarding the length of the single stranded U-stretch, with Hfq requiring a longer polyU tail. They also differ in regard to the sequence upstream of the terminator hairpin, with an A-rich motif, present in ProQ-specific RNAs, disfavouring Hfq binding [64]. Moreover, ProQ interacts with many cis-encoded sRNAs, which are not Hfq targets [63].

ProQ binding to the 3’-end of RNA molecules confers protection from 3’->5’ exonucleases, as many mRNAs are unstable in Δ*proQ* strains. *cspE*, for example, is protected by ProQ from the degradation by the exoribonuclease RNase II [63]. ProQ binding to 3’UTR regions has been also shown to stabilise RNAs and protect them from degradation by polynucleotide phosphorylase (PNPase) in *Neisseria meningitidis*, where the loss of ProQ alters the levels of more than 250 transcripts [67]. Besides conferring protection to RNA molecules from ribonucleases, ProQ can facilitate RNA annealing, as shown for the RaiZ-*hupA* sRNA-mRNA pairing, that occurs near the RBS and thus prevents translation initiation of the mRNA [68].

## CsrA, a riboregulator with a chaperone potential

The protein CsrA (‘carbon storage regulator A’) was initially discovered as a carbon storage regulating protein. Its role in post-transcriptional regulation is determined by interactions with the 5’ UTR and coding regions of many mRNAs, which can result in either activation or repression of their translation. CsrA is the main player of the stationary-phase metabolite control system, but its activity modulates expression of different genes through the entire bacterial life cycle, regulating not only carbon metabolism but also motility, virulence, iron homeostasis, cell envelope integrity and biofilm formation. In *E. coli* CsrA influences metabolite levels [69] and over 700 transcripts that include transcription regulators, ribonucleases and sRNAs. CsrA and its homologues, like RsmA in *Erwinia* spp. (Rsm for ‘repressor of stationary phase metabolites’), are widely distributed in eubacteria. They can be found in gram-positive species, but are mostly distributed within the gram-negative γ-proteobacteria (Table 1). Some members of the *Pseudomonas* genus encode more than one CsrA homologue in their genome [70,71]. In *B. subtilis*, CsrA has been found to assist in base-pairing between sRNA and its target [72].

### Structure

**Figure 4.**
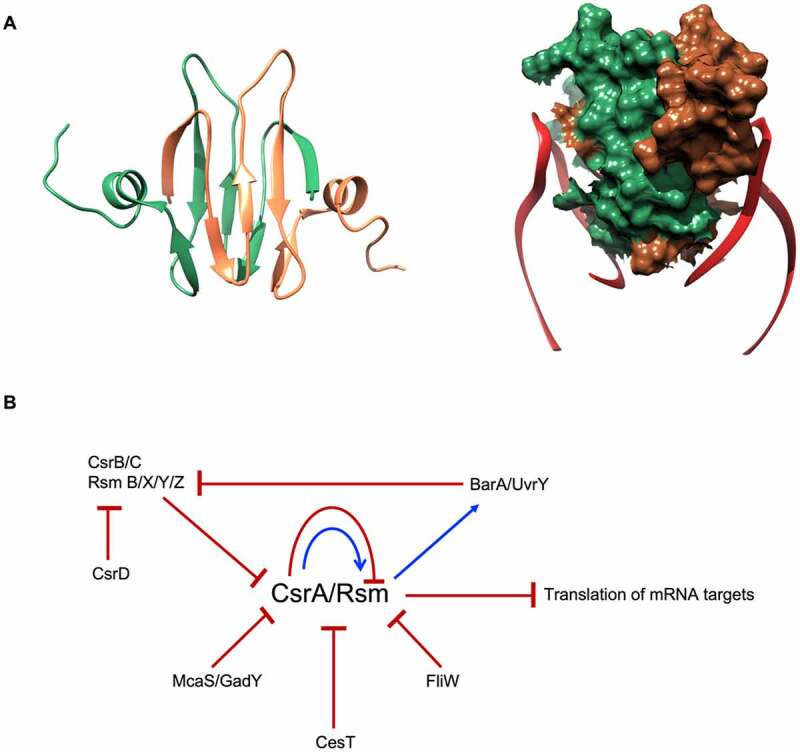
***E. coli* CsrA structure and regulatory circuit**. (A) CsrA forms a dimer (green and brown) with two surfaces for RNA (red) binding. (B) Schematic of CsrA regulation. The expression of *csrA* is regulated on transcription, translation and protein levels: CsrA can regulate its own transcription and translation. It also can be sequestered by sRNAs CsrB/C, GadY and McaS. Moreover, CsrA activity is blocked by proteins CesT in *E. coli* and FliW in flagellated bacteria.

The structure of CsrA revealed a homodimer with each monomer composed of five antiparallel β strands linked with an unstructured C-terminus through a small α-helix [73,74]. The dimer–dimer interface is formed by the two interlocking antiparallel β-sheets (Fig. 4A). The structured part of the protein is well conserved, while the C-terminal unstructured domain shows high divergence [70,71]. Among the Csr/Rsm proteins, RsmN has a truncated C-terminus in *Pseudomonas* spp., yet possesses a short α-helical insert between β strands 2 and 3 that stabilises the dimer [75]. Two RNA fragments, from the same or from different transcripts, can bind to the two opposite sides of Csr proteins, where β_1_ strand of one protomer links with the β_5_ strand of the other, forming a novel RNA binding surface [71,76,77] (Fig. 4B).

### Interaction with RNA

CsrA can bind non-coding RNAs, like CsrB/C and RsmB/X/Y/Z, which mimic target mRNAs, thus acting like CsrA sponges [78]. Sequestering CsrA by these decoys contributes to the Csr control network. CsrB/RsmB and CsrC are about 350 and 245 nucleotides long, respectively, and contain repeated nucleotide sequences with GGA motifs predicted to mimic the Shine-Dalgarno mRNA element that sponge multiple copies of CsrA molecules, thereby antagonising its activity [79–81]. RsmX/Y/Z are shorter, just over 100 nucleotide long RNAs acting via the same mechanism [78,82,83]. CsrB/C sponges form a regulatory circuit together with their target CsrA, which indirectly activates expression of CsrB/C through the BarA–UvrY two-component signal transduction system [80,84]. In *E. coli*, CsrA is regulated also by two small, non-coding RNAs, McaS and GadY, that antagonise CsrA activity [85,86]. Moreover, CsrA activity and RNA binding properties have been shown to be modulated also by proteins: CesT in *E. coli*, which occludes the RNA binding site [87]; and FliW of flagellated bacteria, which regulates CsrA activity allosterically via interaction with its C-terminus [88]. Moreover, CsrA has been shown to autoregulate its own expression through downregulation of translation and through indirect upregulation of transcription of its own gene [89]. In a further level of regulation of CsrA activity, the regulatory protein CsrD controls RNase E/PNPase-dependent degradation of CsrB/C [90] (Fig. 4C).

## A growing appreciation for the previously neglected domains of RNA chaperones

### The C-terminal tails of Hfq

The binding properties and different roles in sRNA-mediated regulation of the surfaces exposed on the hexameric Hfq have been extensively characterised. However, the role of the C-terminal domain (CTD) remains poorly understood. Different studies trying to unravel the functions of this unstructured region have come to contradictory conclusions. While some studies have indicated that deleting this region does not disrupt sRNA-mediated control of gene expression, others have found the CTD critical for this type of regulation [91–94]. Furthermore, the CTD of Hfq does not seem to be required for RNA annealing, but appears to modulate Hfq function by increasing the selectivity of RNA binding, and to aid Hfq recycling by promoting the displacement of RNA duplexes [94].

**Figure 5.**
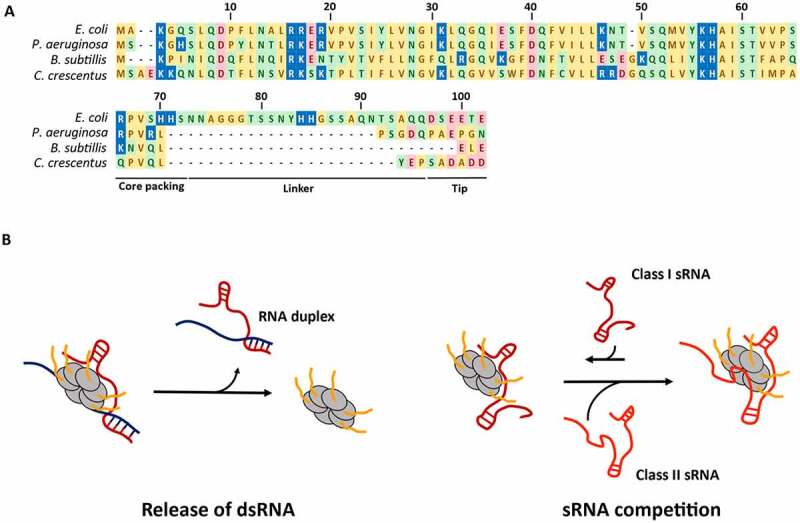
**The C-terminal tails of Hfq: conservation and possible roles**. A) Hfq sequences in different bacterial species. Residue numbering is according to *E. coli* Hfq. Amino acids are coloured according to their polarity (yellow – non-polar, green – polar, red – acidic, blue – basic). Alignment was performed using the MAFFT server at EBI [147]. B) Predicted roles of the Hfq CTD that aid the recycling of the RNA chaperone. Upon RNA duplex formation, the CTD of Hfq facilitates the rapid release of the dsRNA (left). Hfq CTD-rim interactions also increase the selectivity of RNA binding. Through their binding to the proximal and distal faces of Hfq, Class II sRNAs are generally less susceptible to the action of Hfq C-terminal tails and can outcompete the more sensitive Class I sRNAs (right).

The CTD of Hfq is intrinsically disordered and varies in length and amino acid composition across different bacterial species. For instance, the C-terminal tail in *E. coli* spans residues 66–102 and is highly enriched in acidic residues in the tip (-DSEETE), while in *P. aeruginosa* the tail is only 16 amino acids long and has no particular enrichment at the tip. A cross-comparison of over 220 Hfq proteins from different bacterial species divided the CTD into three regions [91]. In *E. coli*, the start of the tail is marked by a strongly conserved proline (P64) followed by a set of residues which also show a high conservation, especially R66, that pack against the core of Hfq. After the core packing region, there is a middle linker region (residues 73–96) which varies highly in length and sequence in different bacteria, and at the very end of the CTD occurs the acidic tip [91] (Fig. 5A).

Competitive binding experiments using a fluorescently labelled CTD peptide have shown that the acidic tip of the CTD could mimic nucleic acids and transiently interact with the rim of the Hfq core [91]. Through this interaction, the CTD competes with RNA molecules for the binding to the core and increases the stringency of substrate selection by Hfq. Truncation of the CTD (Hfq_65_) does not seem to substantially alter the equilibrium dissociation constants of RNA to the proximal and distal faces [94]. In contrast, when compared to the full-length chaperone, Hfq_65_ molecules show an increased affinity for RNA that interact with the chaperone through the rim, pointing to an inhibitory effect of the CTD. When the target RNA binds to the distal face, Hfq–RNA interactions appear to be immune to the autoinhibitory effects of the CTD [94]. The strength and frequency of CTD-rim interactions seem to depend on the number of basic residues on the rim and the local density of acidic residues in the C-terminal tail [91]. Mutating basic residues in the rim of *E. coli* Hfq, R16, R19 or K47, weakens CTD-rim interactions, and changing the acidic residues in the tip of the CTD for non-electronegative ones disrupts them completely [91]. Besides, the length of the linker region also seems to be relevant in determining the rate of CTD-rim interactions, and shortening it results in a higher concentration of acidic residues around the core which leads to an increased autoinhibition by the CTD [91,95].

The capacity of RNA to compete against the CTD for core binding is directly correlated with the length of the nucleic acid and thus the rim appears to have low sequence-specificity [91]. However, the activity of the CTD has a dissimilar effect on RNAs depending on their mode of binding to Hfq. CTD-rim interactions tend to displace Class I sRNAs predicted to interact with Hfq through the proximal face and the rim. On the other hand, the interaction of Class II sRNAs with the distal face of Hfq would make them less sensitive to the action of the CTD, enabling them to resist several annealing cycles. *In vitro* experiments have shown that the capacity of ChiX (Class II sRNA) to outcompete DsrA (Class I sRNA), RyhB (Class I sRNA) and RprA (mixed Class I/II) for the binding to Hfq is negatively affected in Hfq_65_ compared to full-length Hfq [94]. *In vivo*, the removal of the CTD of Hfq leads to a decrease in the accumulation levels of several Class II sRNAs, which lose their kinetic advantage over other RNA molecules, with regard to Hfq binding, and are more rapidly degraded than in the presence of the full-length chaperone [94]. Notwithstanding, the stabilising effect of the CTD does not seem to be restricted to Class II sRNAs, since its truncation also negatively affects the stability of some Class I sRNAs [96]. Additionally, while the CTD does not affect the rate of RNA binding, it is essential upon ternary complex formation for a rapid release of dsRNA that enables the recycling of Hfq [94].

Furthermore, a recent study on the implications of the CTD *in vivo*, has revealed that combinations of mutations in the CTD and the distal face or the rim have synergetic effects which points to a collaboration between the tail and these surfaces on *E. coli* Hfq [96]. For instance, a synergetic effect between distal face mutations and CTD deletion was observed for the sRNA-independent repression of the *mutS* mRNA by Hfq. For distal face function, the core packing region (residues 65–72) seemed to be the only required section of the CTD for Hfq-mediated regulations and the highly conserved R66 appeared to be the most critical residue. Conversely, full-length CTD of Hfq and particularly its tip appears to interact and cooperate with the rim to mediate the regulation of *sodB* by Class I sRNA RyhB.

The determination of the properties that govern CTD-rim interactions poses the question on the role and mode of action of these tails in species in which Hfq has a different number of basic or acidic residues in the core rim or the C-terminal tip, respectively, or in which the length of the linker region in the CTD varies. The analysis of a chimeric Hfq variant in which the core of *E. coli* Hfq was fused to the CTD of *B. subtillis* Hfq (-KNVQLELE), shorter and slightly less acidic than that of *E. coli*, showed that *B. subtillis* CTD contacted the rim more often than *E. coli* CTD, but less strongly and with different amino acid preference [91]. A more recent study focused on *Caulobacter crescentus* Hfq with a CTD which also has an acidic tip (-DADD) but is substantially shorter than that of *E. coli* Hfq (14 residues vs 36 residues). The comparison between the RNA binding activity of the *C. crescentus* variant and that of *E. coli* Hfq showed that the latter had a higher affinity for *E. coli* sRNAs, but also for sRNAs found in *C. crescentus* [95]. When the CTD of *C. crescentus* Hfq was fused to the *E. coli* Hfq core, this chimeric variant had lower RNA binding affinities than the *E. coli* variant, being similar to the binding affinities of *C. crescentus* Hfq. This higher degree of autoinhibition by the CTD of *C. crescentus* Hfq also lead to a stronger inhibition of RNA annealing and could be explained by the higher local density of acidic residues at the tip.

Overall, to date, two main roles on RNA binding have been predicted for the CTD of *E. coli* Hfq. On the one hand, the initial part of the tail is proposed to participate in the function of the distal face of Hfq and mutations in R66 have been shown to affect the binding of Class II sRNAs and of mRNAs to this surface [96] (Fig. 5B). On the other hand, the acidic tip of the CTD has been proposed to mimic nucleic acids and interact with the rim of Hfq, autoregulating the chaperone by competing against non-specific RNA binding and facilitating dsRNA release [91,94,95] (Fig. 5B). Further studies will aid to unveil the reasons behind the overall reduced conservation of the Hfq CTD, as well as other potential regulatory roles this domain plays across different bacterial species.

## The C-terminal domain of ProQ

ProQ and other FinO proteins bind to similar RNA motifs. However, ProQ is able to bind to many RNAs, whereas most of the FinO proteins have a small target pool [63,65]. *Salmonella* FinO, for example, is a plasmid encoded protein that mediates the interaction between the sRNA FinP and the mRNA *traJ* [59,63]. Recent studies show that FinO also regulates another sRNA, RepX, different in sequence but similar in structure to FinP, that originates from the replication control locus of another plasmid. This way, the FinO-encoding plasmid influences the copy number of another plasmid via FinO [65]. ProQ has the C-terminal domain (CTD), that could determine the difference in specificity between ProQ and other FinO proteins, as such extension could directly contribute to the recognition of specific targets [66] (Fig. 3E).

Mutations in the ProQ C-terminal domain do not seem to affect the protein stability [62,97]. Moreover, the CTD appears to be involved in protection from RNase II attack and thus required for RNA stabilisation *in vivo* [62]. The C-terminal domain engagement in RNA binding is still understudied. Two recent studies have investigated the effect of mutations in the C-terminal domain on the function of ProQ in two different pathways in *Salmonella*. The first pathway is the modulation of the succinate-dependent growth *in vivo*, where strains with inactivated ProQ presented a gain-of-function phenotype, with an increased growth rate in the presence of succinate, which when present as a sole carbon source in wild type *S*. Typhimurium causes an extended lag phase and a slowed doubling time [97,98]. The same phenotype was observed for different ProQ mutants, both in the NTD and CTD. However, the CTD mutations had only a modest impact on the ability of ProQ to bind RNA, suggesting that the ProQ CTD might have another role in the control of the succinate phenotype that is not related to the capacity of this protein to interact with RNA [97].

The second pathway is the assembly of the flagellum in *Salmonella enterica*, which is regulated by ProQ via the master regulator of the flagellar gene operon, *flhDC* [62,99–102]. ProQ acts upstream of this regulatory circuit, with an effect on *flhDC* expression, which leads to a lack of activation of flagellar genes transcription [62]. Mutations of the residues G185, T200 and G216, that are exposed on the surface of the C-terminal domain and are conserved in homologous proteins, completely abolished ProQ function in the flagellar system [62] (Fig. 3D).

### Cooperativity between chaperones and other effector moleculesInterplay between Hfq and ProQ

The RNA interactome of Hfq has been widely studied in the past years [46,103–107]. The more recent characterisation of ProQ as an RNA chaperone has also been followed by the characterisation of its RNA targets [58,63]. As discussed above, these two RNA chaperones bind to single-stranded poly(U) tails, frequently found at the 3’ end of sRNAs. Notwithstanding, Hfq has a preference for longer poly(U) stretches and fails to bind some ProQ-specific sRNAs with fewer single-stranded terminal uridines [63,108]. Furthermore, the RNA binding surfaces of Hfq have a higher affinity for certain sequence motifs, while ProQ appears to have a preference for structural motifs. Despite the different RNA binding properties that lead to different RNA interactomes, certain RNA molecules appear to be shared targets of these two RNA chaperones.

In recent studies, Melamed *et al*. have used RIL-Seq to characterise the RNA–RNA interactomes of ProQ and Hfq in *E. coli* [46,47]. These studies have helped to identify novel sRNAs and their targets, revealing RNA–RNA pairs that associate with Hfq and ProQ, in the thousands and the hundreds, respectively. The comparison between both interactomes shows an overlap which accounts for a third of the RNA–RNA pairs bound to ProQ [47]. Of note, out of the 101 shared RNA–RNA pairs, which were generally more abundantly bound to Hfq than to ProQ, the sRNA CyaR was found in 38 of them, including a complex with a region of an operonic transcript that contained the mRNA encoding for Hfq. With regard to the mRNA molecules present in these shared pairs, functional annotation analysis revealed an enrichment for RNAs encoding for outer membranes proteins such as the porins OmpA and OmpC. Despite associating with both chaperones, the interaction of these intersecting RNA–RNA pairs with one of the chaperones does not seem to depend on the presence of the other chaperone since they can also be detected in knockout strains devoid of either ProQ or Hfq. The RNA-RNA pair association with Hfq or with ProQ can have different impacts on the RNA molecules. For instance, RbsZ appears to act as a sponge RNA and decrease the levels of the sRNA RybB in a Hfq-dependent manner. Conversely, the association of this RNA–RNA pair with ProQ results in a protective interaction that prevents RybB downregulation [47].

At a transcriptomic level, Hfq and ProQ seem to cooperate by targeting different sets of genes that allow for optimal regulation of several physiological processes. The effects on the transcriptome of deleting the *hfq* and *proQ* genes in *Salmonella enterica* serovar Typhimurium were recently analysed under conditions that mimic infection [109]. These results showed that both proteins are required for the regulation of certain virulence-related processes such as motility and secretion. The ProQ and Hfq regulons have a reduced overlap during early stationary-phase, but there seems to be certain synergy between both chaperones under infection-relevant conditions as the set of differentially expressed genes in a Δ*hfq* Δ*proQ* strain included genes that were not differentially expressed in either Δ*hfq* or Δ*proQ* strains [63,109,110]. Additionally, processes such as chemotaxis were more severely affected in the double knockout strain than in the individual knockouts [109]. In the plant pathogen *Dickeya dadantii*, deletions of *hfq* and *proQ* result in impaired virulence. Analyses of the expression level of different virulence factors in Δ*hfq* and Δ*proQ* individual and double knockout strains of *D. dadantii* suggested partially overlapping RNA regulons of Hfq and ProQ, which could have cooperative or competing roles. Furthermore, two recent studies in *N. meningitidis* have reported 526 mRNAs targeted by either Hfq or ProQ out of the 2,348 total meningococcal mRNAs detected, from which 41 mRNAs were shared targets [67,111]. With regard to sRNAs, out of the 31 sRNAs bound to either of the chaperones, 6 sRNAs are targeted by both.

Overall, recent transcriptomic analyses in different species and under different physiological conditions have revealed a partial overlap in the regulons and RNA interactomes of ProQ and Hfq [47,67,109,112]. The two major RNA chaperones could have cooperative or competing roles in the regulation of certain physiological processes. Nevertheless, the mechanisms underpinning the interplay between Hfq and ProQ and the extent to which this modulates the post-transcriptional fate of RNA molecules needs further investigation.

## Interplay between Hfq and CsrA

Hfq is known to interact and interplay with other RNA binding proteins than ProQ, for example CsrA. So far, little is known about the functional meaning of the target overlap of these two riboregulators, nevertheless, some examples lead to speculation regarding the biological relevance of the interplay. *hfq* mRNA in *E. coli*, for example, is bound and regulated by both CsrA and Hfq [113,114]. *hfq* mRNA presents only one CsrA binding motif (GGA) at the ribosome-binding site: the binding of CsrA inhibits the translation of Hfq by impeding the ribosome to interact with the transcript without leading to mRNA degradation [114]. How the interplay between the two proteins is regulated and what is the functional role of each RBP binding is still unclear.

Another example is the sRNA Spot42, which is bound both by Hfq and by CsrA [115,116]. Spot42 post-transcriptionally represses operons that facilitate metabolism of non-preferred carbon sources. The synthesis of Spot42 is repressed by transcription factor CRP when activated by cyclic AMP and is induced in the presence of glucose. One target of Spot42 is the *srlA* transcript, which encodes a component of a d-sorbitol-specific phosphotransferase system. When Spot42 binds *srlA*, it negatively regulates the translation of *srlA* mRNA in a Hfq-dependent manner. CsrA has been recently shown to bind Spot42, preventing cleavage of the small RNA and leading to an enhanced repression of *srlA*. Furthermore, Spot42 is not as efficient as CsrB and CsrC in recruiting CsrA, so there is potential for graded effects depending on the relative abundance of these factors. The discovery of the interaction with the Spot42 has opened tantalising possibilities for a new role and mechanism of action of CsrA [115,116].

Finally, in *P. aeruginosa* the RNA-binding protein RsmA binds to nascent transcripts [117], like *hfq* [118]. This co-transcriptional binding of RsmA could prevent the transcription terminator factor Rho from accessing a loading site on the mRNA. Furthermore, RsmA and Hfq have an extensive overlap in the RNA targets, as it was shown that both Hfq and RsmA bind to polycistronic (*estA*) and monocistronic mRNAs (*amrZ*). By binding to the same transcript, RsmA and Hfq could act in combination to exert control on translation and abundance of transcripts in the cell: if RsmA and Hfq are sensitive to different environmental stimuli, this would enable the control of common transcripts in a synchronised way that reflects the effects of both chaperones [117].

## Hfq interaction with Crc

**Figure 6.**
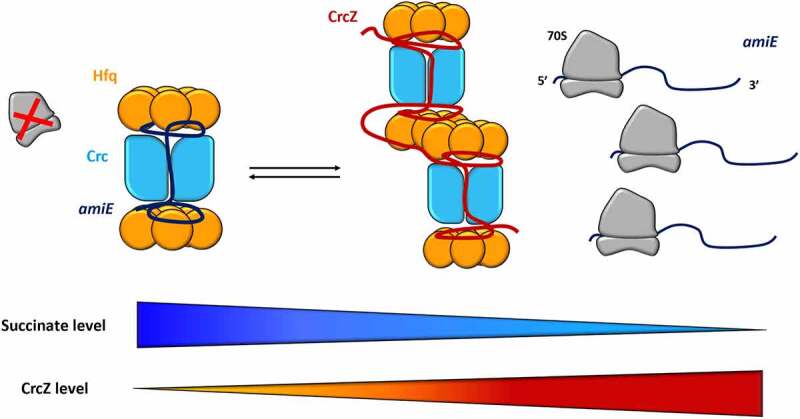
**Hfq-Crc cooperation in *Pseudomonas aeruginosa***. When succinate, the preferred carbon source, is available, Hfq and Crc bind to the mRNA target, *amiE*, masking the ribosome-binding site and preventing its translation [119,120]. The distal face of captures ARN-rich repeat motifs near the RBS, where A- and R-bases occupy basic pockets on the Hfq distal side, leaving the RNA backbone and N-bases exposed to Crc. In contrast, when succinate levels are low, CrcZ, an sRNA rich in ARN-motif, is expressed to sequester Hfq away from substrate RNAs, such as *amiE*, allowing ribosomes to bind and begin translation [119].

A well-studied example of cooperative targeting of transcripts can be found in the main Carbon Catabolite Repression (CCR) regulon in *P. aeruginosa*, i.e. the Hfq-Crc mediated translation repression system [118,119]. CCR ensures that alternative nutrients are not utilized until the preferred carbon source of *P. aeruginosa*, succinate, is depleted. When succinate is available, Hfq and Crc (catabolite control protein) mask ribosome-binding sites on target mRNA transcripts to prevent translation of genes involved in the uptake and metabolism of secondary carbon sources [119,120]. Recent cryogenic electron microscopy (cryo-EM) studies of Hfq-Crc complexes on a short octadecameric segment derived from the 5’ upstream untranslated region (5’ UTR) of *amiE* mRNA revealed for the first time how Hfq captures and presents substrate RNAs to Crc during CCR [27] (Fig. 6). In particular, the Hfq distal side captures ARN-rich repeat motifs near the RBS, which then recruits Crc. The A- and R-bases occupy basic pockets on the Hfq distal side, while the RNA backbone and N-bases are exposed to Crc. Although no binding affinity was observed between Crc and free RNA molecules, Crc engages the Hfq-RNA intermediate to sequester the RBS from ribosomes and prevent expression of *amiE* and more than 100 other metabolic mRNAs [27,118,121]. Single-molecule fluorescence assays and molecular dynamics simulations showed that the Hfq/RNA intermediates are transient, but that Crc shifts the equilibrium towards assemblies with increased stability and effectiveness [28,122]. Notably, multiple hexameric Hfq and Crc molecules participate in these translation repression complexes, giving rise to polymorphic, RNA sequence- and RNA fold-specific higher order complexes through cooperative assembly (Dendooven et al. 2021). Upon exhaustion of the preferred carbon source, the ARN-rich sRNA CrcZ is expressed and sequesters Hfq away from substrate RNAs, alleviating CCR [119] (Fig. 6). How CrcZ competes with the substrate RNAs for Hfq binding is a puzzle, but molecular dynamics simulations on the Hfq-*amiE* intermediate assembly by Krepl *et al*. revealed a putative mechanism for rapid cycling of RNAs on the Hfq distal side. Local perturbations in the form of *syn-/anti* flipping of the A-site base in its distal Hfq pocket could allow for rapid exchange for of RNAs on the Hfq surface, and more research will further explore this [28].

## Hfq-RNase E

RNase E is a key enzyme for RNA metabolism in many bacteria. It belongs to the RNase E/G enzyme family, with members present across the proteobacteria, actinobacteria and firmicutes phyla, and with homologues also found in cyanobacteria and plant chloroplasts. RNase E is an endoribonuclease catalysing the first RNA cleavage that initialise a complete degradation of transcripts [123,124]. However, it also participates in the maturation of certain RNA molecules, being involved in processing of structured RNA precursors. The catalytic activity is localised to the N-terminal domain of RNase E, which forms a homotetramer with an unstructured C-terminal domain extending from each protomer [124,125]. In *E. coli* and many other bacteria, several of the key enzymes involved in RNA processing and degradation assemble to form a central multi-enzyme machinery in the cell, known as the RNA degradosome [126].

**Figure 7.**
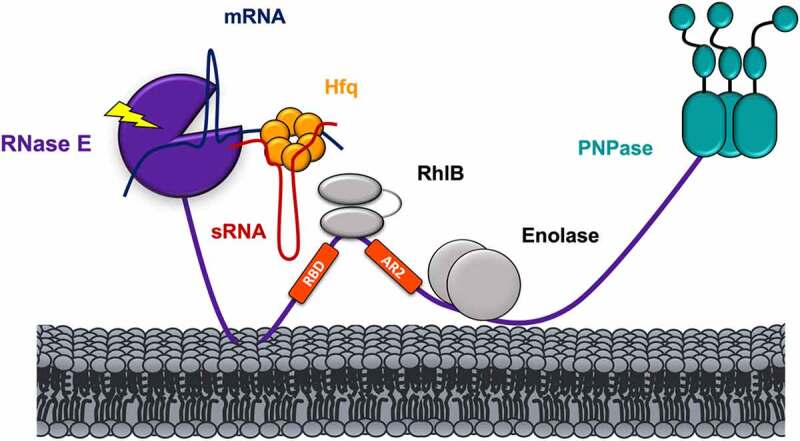
**Model of Hfq-RNase E interaction in *E. coli***. The interaction between Hfq and RNase E is RNA-mediated. The ternary complex formed by Hfq-sRNA-mRNA interacts with two RNA binding regions on the C-terminal domain of RNase E, namely RNA-binding domain (RBD) and second arginine-rich region (AR2) labelled in red [37]. The CTD of RNase E holds the ternary complex in positions and facilitate the delivery of the mRNA target to its catalytic core. Additional components of the *E. coli* degradosome (i.e. RhlB, enolase and PNPase) are also shown.

The potential interplay between Hfq and the degradosome has been postulated in many studies. The interaction between RNase E and Hfq is RNA-dependent, and the two proteins are unlikely to directly interact *in vivo* in the absence of RNA molecules [37,41,127–129]. Other proteins involved in RNA metabolism, such as RNA polymerase, polyA polymerase and Rho transcription termination factor, also interact with Hfq in an RNA-mediated manner [128]. Moreover, sRNA-Hfq-mRNA ternary complexes guide RNase E cleavage, and, when sRNAs are 5’-monophosphorylated, they activate the catalytic core of RNase E triggering the degradation of the target [127]. RNA could bridge between Hfq and the flexible recognition core of the RNA degradosome, comprising of a fragment of the RNase C-terminal domain including two RNA binding sites, and the binding sites for RhlB and enolase with the associating proteins [37]. This complex could be the mediating hub for RNA substrate recognition that transfers the signal to the RNase E NTD to invoke the cleavage (Fig. 7). sRNA-Hfq binding to the recognition core does not displace either RhlB or enolase, hinting a possible function of these two enzymes in this pathway [37]. The matchmaking abilities of Hfq are the foundation of the sRNA mediated target degradation, emphasizing a fundamental role of this chaperone in the post-transcriptional gene regulation.

## Hfq-PNPase

PNPase is a conserved, ancient exoribonuclease that in bacteria processively degrades RNA molecules from the 3’ end either in isolation or in conjunction with the RNA degradosome. However, PNPase is more than just an RNA degrading enzyme, and its multiple functions include stable RNA processing and polymerisation of heterogeneous tails on existing RNA molecules [130]. The repertoire of PNPase activities has been recently shown to be larger than anticipated, as it can also serve as a chaperone for some RNA species when in conjunction with Hfq.

**Figure 8.**
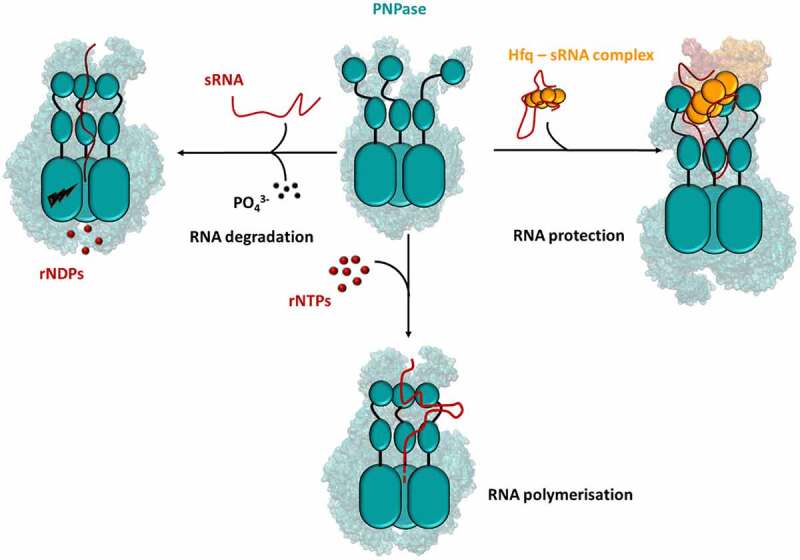
**Hfq forms a protective complex with the exoribonuclease PNPase**. In *E. coli*, PNPase (teal) can act as a conditional chaperone when bound with sRNA and Hfq (Orange; right panel) [131]. In the absence of Hfq, PNPase can either degrade RNA (left panel) or add heterogeneous tails to existing RNA molecules (bottom panel).

Deletion of PNPase causes increased stability of many transcripts, but, surprisingly, also destabilization of several sRNAs. The recent characterisation of the structure of a PNPase-sRNA-Hfq ternary complex by cryo-EM helped to understand the interactions underpinning this phenomenon [131] (Fig. 8). PNPase, as an indispensable component of this assembly, participates in the chaperoning of the captured RNA molecules, conferring stability and protection from other cellular ribonucleases like RNase E.

The PNPase-sRNA-Hfq complex brings together PNPase and Hfq. However, no contacts are made between these proteins and the ternary complex is maintained solely by interactions of both proteins with the same RNA molecule. This allows for some flexibility in the formation of protective complexes of this kind. In these assemblies, the sRNA interacts only with the RNA binding domains of PNPase, S1 and KH, being rerouted away from the catalytic core, where the active sites of the enzyme localise. Hfq binding on the other side, sandwiches the sRNA between both proteins and makes it to some extent inaccessible to other cellular components. As such, the Hfq-sRNA intermediate hijacks the PNPase RNA binding modules (KH-S1) for downstream regulatory processes (e.g. pairing with a target RNA) and bypasses the catalytic core entirely. Within this complex, however, the RNA must still be able to pair with its target transcript. Thus, mechanisms must be in place to allow for relaxation of the assembly when the target mRNA is encountered, although these are still to be characterised.

## Other RNA chaperones

Besides Hfq, ProQ and CsrA, other proteins with RNA chaperoning function can be found in bacteria, some of which are limited to certain RNAs, while others act on larger RNA pools. Among these proteins, cold shock proteins have an important role. RNA molecules have a natural ability to adopt more than a single stable conformation, and the adoption of a certain conformation depends on environmental conditions. Upon cold shock, the structure of RNA changes as with the drop of the temperature many RNA structures become more stable, changing RNA accessibility and fold. Under these conditions, cold shock proteins (Csp) exert their chaperoning activity and melt RNA secondary structures, restoring processes such as transcription and translation [132]. In *E. coli*, the main cold shock protein is CspA, and nine proteins from the CspA family have been identified, named CspA to CspI. Homologues of CspA are widespread in prokaryotes, acting in response to many different environmental stresses [133,134].

The CspA fold is structurally similar to the fold of the S1 domain, an RNA binding domain identified in many proteins involved in RNA metabolism [133]. Six copies of this domain are present in the ribosomal S1 protein, which has also been found to have chaperoning capabilities and destabilise secondary RNA structures [4,135]. S1 is the only ribosomal protein not stably associated with the ribosome and has been shown to participate in the binding and remodelling of mRNAs according to translation requirements [4]. Moreover, ribosomal proteins in general are able to chaperone RNA molecules: for example, protein S12 was shown to be capable of facilitating RNA-based reactions, probably by optimising RNA conformation [5]; and almost a third of proteins of the large ribosomal subunit were found to have chaperoning activity *in vitro* [136]. Another bacterial protein, StpA, identified in *E. coli* as a homologue of the histone-like protein H-NS, was shown to be able to act as a non-specific RNA chaperone [137]. However, no *in vivo* substrate of this protein has been identified so far. StpA participates in the regulatory circuit of *ompF* expression by modulating the stability of the sRNA MicF that represses *ompF* translation [138]. StpA chaperoning activity depends on its C-terminal domain, which is capable of RNA refolding *in vitro* [139].

## Concluding remarks

RNA fulfils a broad range of functions in the cell: from encoding the amino acid sequence in proteins, through acting as a building block for many molecular assemblies, catalysing reactions, transducing cellular signals, to being a regulator of many cellular processes. This versatility has arisen in the face of the labile character of RNA molecules, their chemical instability and susceptibility to ribonuclease attacks. Furthermore, RNA molecules can adopt more than one stable conformation and often get trapped in folding intermediate states. RNA chaperones can prevent or resolve such trapped states without requirement for ATP consumption. Instead, they simply bind and release an RNA molecule so that misfolded regions are destabilised, and the structural confinement resolved. They can also help in annealing two RNA molecules and stabilising bound RNA substrates to support their function [140].

RNA chaperones constitute a very diverse group and are widespread across all domains of life. On the one hand, the list of bacterial proteins with chaperone activity is constantly growing. For example, three small proteins FbpA, FbpB and FbpC in *B. subtilis* were shown to act as chaperones [141], not to mention the recent discovery of a chaperoning mode of PNPase [131]. Moreover, in gram-positive bacteria that lack Hfq or ProQ homologues, such as *Streptococcus pneumoniae*, proteins with a KH domain, like KhpA and KhpB, could act as RNA chaperones [142–144]. On the other hand, the ones that have been known for decades, seem to have an incredibly versatile repertoire of regulatory activities across bacterial species which can expand beyond their interactions with RNA, as shown for Hfq. This pleiotropic regulator has been very recently found to be fundamental for the silencing of prophages and mobile genetic elements by binding to these DNA regions and driving the formation of phase-separated condensates and of heterochromatin-like domains [145].

Also increasingly apparent is the expanded network of cooperation and interplay between RNA chaperones and between the chaperones and other proteins involved in RNA metabolism. The recent discovery of the overlapping targetome of Hfq and ProQ suggests that these most common RNA chaperones could be able to support the same pathways or compete for their targets depending on the circumstances [47,112,146]. Although the rivalry is a possibility, cooperation is something observed more often thus far. The synergy seems to be a concept between the Hfq-RsmA interplay that involves regulation of the expression of the same RNA molecule [117], as well as between Hfq and Crc during regulation of carbon metabolism in *P. aeruginosa* [27]. Moreover, Hfq cooperation with RNase E in sRNA-mediated gene regulation and with PNPase in stabilisation of many sRNAs underlines the importance of protein networking in order to extricate all the functionality encoded in the RNA.

Although the field is mature, many questions remain unanswered, e.g. how does a chaperone find its RNA target *in vivo*? How does it detect if the RNA structure is right? What is the explanation of the large discrepancy between the number of RNA chaperones in the cell and the number of RNA targets? When is a chaperone-bound RNA fully released? How is the RNA delivered to its destination? How does the pool of RNAs get accurately divided between cellular chaperones? How do the chaperones manage to coordinate their RNA targets without creating chaos by cross-interference? Despite some hints from bacterial physiology that indicate spatial separation of different processes in the cell or characteristics of RNA substrates bound by a particular chaperone, clear answers to these questions are yet to come.
